# Current and Emerging Therapeutic Options in Adrenocortical Cancer Treatment

**DOI:** 10.1155/2012/408131

**Published:** 2012-08-14

**Authors:** Antonio Stigliano, Lidia Cerquetti, Camilla Sampaoli, Barbara Bucci, Vincenzo Toscano

**Affiliations:** ^1^Endocrinology, Department of Clinical and Molecular Medicine, Sant'Andrea Hospital, Faculty of Medicine and Psychology, “Sapienza” University of Rome, Via di Grottarossa, 1035, 00189 Rome, Italy; ^2^Research Center, San Pietro Hospital Fatebenefratelli, Via Cassia 600, 00189/Rome, Italy

## Abstract

Adrenocortical carcinoma (ACC) is a very rare endocrine tumour, with variable prognosis, depending on tumour stage and time of diagnosis. The overall survival is five years from detection. Radical surgery is considered the therapy of choice in the first stages of ACC. However postoperative disease-free survival at 5 years is only around 30% and recurrence rates are frequent. *o,p'*DDD (*ortho-, para'-,* dichloro-, diphenyl-, dichloroethane, or mitotane), an adrenolytic drug with significant toxicity and unpredictable therapeutic response, is used in the treatment of ACC. Unfortunately, treatment for this aggressive cancer is still ineffective. Over the past years, the growing interest in ACC has contributed to the development of therapeutic strategies in order to contrast the neoplastic spread. In this paper we discuss the most promising therapies which can be used in this endocrine neoplasia.

## 1. Introduction

Adrenocortical carcinoma (ACC) is a rare malignant disease with poor prognosis and an estimated incidence between 1 and 2 per million population annually [1–4]. The age distribution is reported as bimodal with a first peak in childhood and a second higher peak in the fourth and fifth decade [3, 4]. Genetic studies performed on ACC were focused on molecular alterations either at the germline level in rare familial diseases or at somatic level in sporadic tumors. These advances underline the importance of genetic alterations in ACC development and indicate various chromosomal regions (2, 11p15, 11q, 17p13) and genes (IGF-II, p53, **β**-catenin, etc.), potentially involved in ACC [5–11]. In particular, the monoclonality analysis indicates that tumor progression is the final result of an intrinsic genetic mutation, whereas polyclonality suggests that tumor cells are affected by local or systemic stimuli. Analysis of the pattern of X-chromosome inactivation in heterozygous female tissue shows that ACC consists of monoclonal populations of cells [12]. Molecular alterations lead to inactivation of the tumor suppressor genes and sequential activation of the oncogenes. The insulin growth factor II (IGF-II) system, located at 11p15, is heavily involved in ACC ethiopathogenesis [5]. Loss of heterozygosity (LOH) at chromosome region 11p15, associated with a higher risk of tumor recurrence, is more frequent in ACC than in adrenal adenomas [6]. The development of ACC may be due to an activation of the Wnt signaling pathway caused by germline mutations of the Adenomatous Polyposis Coli (APC) gene [7, 8]. Germline mutations in TP53 are identified in 70% of families with the Li-Fraumeni syndrome. This syndrome shows dominant inheritance and confers susceptibility to ACC and other tumor development [9, 10]. LOH at 17p13 of p53 has been consistently demonstrated in ACC but not in adrenocortical adenomas [6, 11]. The diagnosis of malignancy of adrenocortical tumors relies not only on careful clinical and biological investigations, but above all on improved of radiological imaging such as computerized tomography (CT) or magnetic resonance imaging (MRI) and more recently the use of  ^18^F-FDG PET to distinguish between benign and malignant lesions [13, 14]. Patients could present signs or symptoms of steroid hormone excess or signs due to the presence of abdominal mass. Hormonal investigations demonstrate cortisol oversecretion in most ACCs. Some of these have a cosecretion of glucocorticoid and androgens. Androgen secreting ACCs in women induce hirsutism and virilization. On the contrary, estrogen-secreting adrenal tumors in males lead to gynecomastia and testicular atrophy [15]. High level of DHEA-S is another marker suggesting ACC, whereas decreased serum DHEA-S concentrations are suggestive of a benign adenoma [15]. ACC producing aldosterone is very rare and present with hypertension and pronounced hypokalemia [16]. Hormonally inactive ACCs usually present with gastrointestinal symptoms or back pain caused by a mass effect of the large tumor.

ACCs show variable prognosis, depending on tumour stage and time of diagnosis with a median overall survival longer than 5 years for patients with stage I and stage II, whereas in stage III and IV, it decreased [17, 18]. Frequency of metastasis associated with ACC varies depending on the study, ranging from 30% to 85% of patients with distant metastasis at the time of presentation [19].

The classification of ACC by the *International Union against Cancer* (UICC) and the *World Health Organization* (WHO) in 2004 is based on the *tumor, lymph node and metastasis *(TNM) criteria as described by Macfarlane [20] and later modified by Sullivan et al. [21]. Thereafter in 2008, the European Network for the Study of Adrenal Tumors (ENS@T) proposed a revision of this staging, in which stage III is defined by tumour infiltration in surrounding tissue or tumour thrombus in vena cava/renal vein or positive lymph nodes, and stage IV is defined only by the presence of distant metastases (Table 1) [22]. The ENSAT-staging system showed higher accuracy in predicting cancer-specific mortality risk than the 2004 UICC-staging system in the ACC prognosis (83% versus 79.5%). This is currently the best criteria staging of ACC [17, 23].

Unfortunately, ACC prognosis is very poor. It depends largely on the stage of tumor: the rate of survival at 5 years is estimated at 60% for patients with stage I cancer, 58% for stage II tumors, 24% for stage III tumors, and 0% for disease stage IV [24].

Patients with stage I and II of disease are amenable to potentially curative surgery. They had a prolonged survival compared to patients with stage III and IV [24]. Macfarlane [20] reported a median survival of 2.9 months for untreated carcinomas, whereas between 16% and 38% of those treated have a median survival at 5 years, depending on the series studied [25]. Patients with stage I and II of disease have a similar prognosis, which is much better than that seen in stage III and IV [22]. Median overall survival rate is 38% and 50%, respectively, for patients undergoing surgery [23]. The median survival in metastatic disease, however, is almost always less than 12 months from time of diagnosis [17]. Factors indicative of good prognosis are early diagnosis of disease (stages I and II) and complete tumor resection (R0).

However, available clinical series shows that diagnosis usually occurs in advanced stages of disease [25–27]. The complete surgical resection of the tumor, the diameter of the lesion, the secretory activity of the tumor, and some molecular markers are factors that can influence the prognosis. Significant reduction in survival is associated with ACC greater than 12 cm in diameter, even if completely resected [28, 29]. Cancer-producing cortisol excess or a mixture of cortisol and androgens, is reported to be worse compared to ACC secreting androgens alone or hormonal precursors [24, 30, 31]. Overexpression of IGF-II and topoisomerase 2A (TOPO 2A) [32] and loss of heterozygosity (LOH) on chromosome 11p15 and 17p13 loci [6] were identified as factors could potentially be used to predict a grade of malignancy. However, the prognostic value of most of these markers has not yet been established by prospective studies [29].

Therefore, the clinical picture and the prognosis of patients affected by ACC appear to be rather disappointing. Following recent data acquisition, it is now well-established opinion that ACC requires a multidisciplinary management. The crucial first therapeutic step is radical surgery, also in the incidence of isolated metastatic disease [2, 28, 33]. However, the most widely used medical therapy for patients unsuitable for surgery is treatment with mitotane, an insecticide derivative *o',p'*-DDD (*ortho, para'*, dichloro-, diphenyl-, dichloroethane) either alone or in combination with chemotherapeutic agents [24, 29, 30, 34–40]. Unfortunately, given the high toxic effects resulting from mitotane therapy, the response rates are rather low in ACC [25, 36, 41]. Several cytotoxic pharmacological agents, such as cisplatin, etoposide, doxorubicin/adriamycin, vincristine, 5-fluorouracil, and streptozotocin, have been used individually or in a combination regimen in the treatment of patients with late-stage ACCs [36, 41–43]. To date, the studies that have shown the highest rates of therapeutic response were the so-called “Italian” protocol, consisting of etoposide, doxorubicin, and cisplatin, with concomitant mitotane administration (EDP/M) [29]. A second active regimen is the combination of streptozotocin and mitotane [37]. These two therapeutic regimens were tested in the first randomized controlled trial Phase III started in 2004 for ACC (FIRM-ACT: First International Randomized Trial in Locally Advanced and Metastatic Adrenocortical Carcinoma Treatment). The trial was started in 2004 in order to establish the gold standard in the treatment of locally advanced adrenal cortical carcinoma not amenable to surgical resection (resp., stages III and IV) and in patients with a poor life expectancy. The growing interest in this neoplasia has encouraged molecular biology and pharmacology studies. In recent years research efforts have focused on the identification of molecular biomarkers of this neoplasia. The purpose of these studies is to improve the diagnostic and therapeutic options in ACC. The aim of this paper is to describe the current treatment options in patients with ACC and establish innovative strategies for this cancer.

## 2. Surgery

Complete surgical removal of ACC represents the current treatment of choice for this tumor. The likelihood of achieving a healing is by radical surgery, especially in the lower stages of cancer (I and II). A disease-free resection margin (R0) is also an important predictor of long-term survival. However, locoregional recurrence or the appearance of distant metastases during the subsequent followup is common (85%) even after complete resection of the tumor [17, 22, 24, 33, 35, 44–46]. The probability of failure increases in advanced stage of disease. This happens when the lesion is greater than 12 centimeters in maximum diameter, with a high mitotic rate and intralesional hemorrhage [19, 47]. Therefore surgery is demanding and must be performed by a highly experienced surgical team using a laparotomic approach. The utmost care should be taken in order to reach a negative resection margin (R0) and to avoid tumor spillage in the abdominal cavity during its removal since this is unfavorable prognostic factor. The role of laparoscopy in the removal of an ACC has not yet been defined. Its influence on the prognosis of the disease is still unspecified, and there are no randomized trials which have compared the efficacy of laparotomic adrenalectomy versus laparoscopic adrenalectomy [29]. Recently, the role of laparoscopy in the surgical treatment of the ACC has been much debated. Some studies have shown that there was no difference in the oncologic outcome between the laparoscopic and the laparotomic approach in patients affected by ACC [48, 49]. Retrospective data from the German ACC Registry shows that the removal of regional lymph nodes significantly reduced tumor recurrence and disease-related death in patients with localized ACC [50].

Currently the laparotomic approach is considered more reliable in the instance of preoperative diagnosis of ACC. Due to the frequent invasion or the close adhesion of the ACC to adjacent organs, surgery often requires excision en bloc of the ipsilateral kidney, spleen, and a partial pancreatectomy in the case for left adrenal cancer and a partial hepatectomy for right adrenal cancer. Furthermore, removal of the abdominal lymph nodes is performed to remove ACC [29].

Metastatic disease debulking surgery, which removes as much of the tumor as possible, helps to reduce the blockage caused by a mass, usually large as well as the hormonal excess produced by the tumor. However, there is no significant effect of this therapy, since the median survival of patients with incomplete resection of the primary tumor or inoperable metastatic disease not removable by surgery appears to be less than 12 months [24, 33]. Surgical resection of recurrent disease is accepted as a treatment option of choice, as it is associated in a different series with an increase in median overall survival. However, a complete cure was rarely achieved. The surgical approach includes the removal of locoregional tumor recurrence and excision of isolated metastatic foci in the liver and lung [28].

## 3. Mitotane Treatment

Mitotane (1,1-dichloro-2-(*o*-chlorophenyl)-2-(*p*-chlorophenyl)-ethane or *o',p'*-DDD), is an isomer of the insecticide DDT. The first description about its use was made in 1948 when it was shown to produce adrenal atrophy in dogs [51]. This drug acts by inhibiting 11*β*-hydroxylation and P450 side chain cleavage in the mitochondria of steroidogenic cells, therefore blocking cortisol synthesis, decreasing both plasma and urine steroid levels [52]. Since then, mitotane has been used in the treatment either in the first or in advanced stages of ACC [34–40]. Mitotane exerts a specific cytotoxic effect on adrenal cortex cells, resulting in focal degeneration of the fasciculata and reticularis areas while the effects on glomerular are relatively scarce [53]. The effect of this adrenolytic drug is due to its metabolic activation via a hydroxylation reaction. Dehydrochlorination occurs with the production of an acylchloride. This reactive compound may bind covalently to intracellular macromolecules, and in particular to mitochondrial proteins, exerting their biological activity, or become the main inactive metabolite of mitotane (*o,p'*-DDA). Drug activation occurs primarily within the mitochondria in the adrenal cortex cells, while a small portion is subjected to the action of hepatic microsomal enzymes. Metabolic activation of mitotane in the liver induces the formation of two metabolites 1,1-(*o,p'*-dichlorodiphenyl)-2,2-dichloroethane (*o,p'*-DDA) and 1,1-(*o,p'*-dichlorodiphenyl) acetic acid (*o,p'*-DDE), deriving from *β*- or *α*-hydroxylation of *o,p'*-DDD respectively [54]. It has been demonstrated that *o,p'*-DDA represents the active metabolite of mitotane because the *β*-hydroxylation of *o,p'*-DDD results in an adrenolytic effect while the *α*-hydroxylation of *o,p'*-DDD results in a deactivation. On this basis it was hypothesized that the measurement of o,p'-DDA, in patients affected by ACC, can predict the response to mitotane [55]. The effect of increased hepatic metabolic activity is reflected in the reduction of the main urinary catabolite of cortisol and the increasing production of water-soluble polar metabolites of cortisol [39]. The effect of mitotane on the pharmacokinetics of other drugs is not fully understood. Recently, van Erp et al. have observed that mitotane was capable of inducing the activity of hepatic CYP3A4 potentially interfering with the therapeutic efficacy of other molecules including antineoplastic drugs [56], since many drugs are metabolized by CYP3A4. This aspect will need to be considered in the treatment of patients with ACC and in the future design of clinical trials involving the use of chemotherapy in combination with mitotane.

Furthermore, mitotane inhibits the production of testicular androgens, acting as an antagonist on the progesterone and androgen receptors and as an agonist of the estrogen receptor [39]. Beside its adrenolytic effects, mitotane inhibits MDR-1/P-glycoprotein, a multidrug resistance protein, thus enhancing the effect of different chemotherapy drugs [30, 57, 58]. The drug is administered orally at a dose generally greater than 4 g/day, taking the therapeutic window between 14 and 20 *μ*g/dL into account [35, 59, 60]. The plasmatic half-life is approximately 2-3 hours, gradually increasing from the beginning of therapy in relation to drug accumulation in adipose tissue [61]. The daily amount of drug absorbed is excreted in the urine as inactive metabolites, mainly in the form of *o,p'*-DDA, a lower percentage, however, is excreted in the feces. As mitotane accumulates in adipose tissue, the plasma elimination half-life is extremely long (18–159 days) [62].

In patients treated with mitotane, it is essential to establish replacement therapy with glucocorticoids (preferably hydrocortisone 50 mg/day) for the effect on the suppression of cortisol synthesis and the increase of its peripheral catabolism [39]. The major limitation in the use of this drug is linked to the appearance of significant side effects affecting the gastrointestinal tract with the onset of anorexia, nausea, vomiting, and diarrhea and CNS with lethargy, drowsiness, depression, vertigo and ataxia [17, 63]. In light of these findings, ACC-affected patients taking mitotane must be periodically subjected to the plasmatic assay of drug concentration. In addition, it is appropriate to have a close followup with clinical and diagnostic monitoring of liver function.

Therefore mitotane is a therapeutic indication in the treatment of inoperable ACC and in preparing for adrenalectomy. However, the high rate of recurrence of ACC justifies its use in adjuvant therapy following surgical resection [40, 64–66], in accordance with the concept of adjuvant therapy, providing the drug administration immediately after surgery [67]. The different considerations on the effectiveness of adjuvant therapy can be justified by the hypotheses that patients affected by ACC vary in their ability to metabolic drug transformation [37]. Currently, to evaluate the effectiveness of adjuvant therapy with mitotane, a randomized prospective study, called ADIUVO (http://www.adiuvo-trial.org/), is ongoing. This controlled trial provides the enrollment of patients randomized to mitotane treatment after ACC complete resection or followup without drug treatment.

## 4. Chemotherapy

Chemotherapy drugs alone or in combination with mitotane are employed due to the high aggressiveness and poor prognosis of ACC, especially when not amenable to surgical resection or when present in metastatic stage. This combination exploits the ability of mitotane to overcome the drug-resistance induced by P-glycoprotein, which is widely expressed in ACC. The chemotherapeutic agents used individually or in a combination regimen in the treatment of patients with ACC in advanced stages include cisplatin, etoposide, doxorubicin/adriamycin, vincristine, 5-fluorouracil, and streptozotocin [29, 30, 36–39]. Although the results are variable, there is some evidence that cisplatin, alone or in combination with etoposide, exerts a favorable therapeutic effect against ACC at an advanced stage. Bukowski et al. [36] evaluated the effectiveness of the combination of cisplatin with mitotane, achieving a complete response to therapy of 30%. Bonacci et al. [42], using a regimen that included the combination of cisplatin, etoposide, and mitotane, achieved an overall response of 33% while Burgess et al. [43], using a combination of cisplatin and etoposide without mitotane, obtained a response rate of 46%. Although Williamson et al. [41] administered the same protocol (cisplatin plus etoposide) without mitotane to patients with unresectable or metastatic ACC, they achieved a complete response of less than 11%.

To date, the studies that have shown the highest rates of therapeutic response were those performed by Khan et al. [37], and Berruti et al. [30] administered the combination of streptozotocin and mitotane and the combination of etoposide, doxorubicin and cisplatin (EDP) in repeated cycles, in association with mitotane, respectively (Table 2). In Berruti's study, which was based on a large prospective multicenter phase II trial, the EDP combination with mitotane was administered to 72 patients affected by ACC not amenable to surgery: 5 patients achieved a complete response to therapy and 30 a partial response, giving an overall response rate of 48.6%. Khan's study, instead, evaluated the effectiveness of the combination of streptozotocin and mitotane in 22 patients with ACC, giving an overall response of 36.4% (1 patient with complete response and 7 with partial response). In light of these results, the International Consensus Conference on Adrenal Cancer of Ann Arbor recommended the use of these protocols as first-line regimens against metastatic ACC in 2003 [29]. The First International Randomized Trial in Locally Advanced and Metastatic Adrenocortical Carcinoma Treatment (FIRM-ACT) (http://www.firm-act.org/) was initiated in April of 2004, in order to establish the gold standard in therapy in advanced ACC not amenable to radical surgical resection (resp., stages III and IV), in patients with a life expectancy greater than 3 months. The FIRM-ACT was therefore initiated in order to make a comparison with Berruti et al. and Khan et al., chemotherapeutic protocols. The study provided the randomized administration of a combination EDP-mitotane every 4 weeks and streptozotocin-mitotane every 3 weeks in patients with advanced ACC. Patients with disease progression were treated with the alternative combination therapy. The primary objective of the trial was to compare the survival rates. Secondary objectives consisted of evaluating the quality of life, time to disease progression, response rate to therapy, duration of response, and disease-free survival in the two-regimen protocols. The FIRM-ACT study is now completed and the results recently published have shown that patients treated with EDP-mitotane combination had a significantly higher response rate than those treated with streptozotocin-mitotane. Unfortunately, no differences were observed in overall survival confirming the poor prognosis in patients affected by advanced ACC. No differences were found in the quality of life and adverse events in patients receiving the two therapeutic regimens [68].

## 5. Radiotherapy

The efficacy of radiotherapy in ACC has been largely debated. This neoplasm has indeed considered radio resistant for a long time, and many authors have noticed poor results in patients subjected to radiotherapy after surgical removal of the adrenal mass [67, 69]. In other studies, however, a response rate of approximately 42% of cases has been described [17]; moreover, it has also been demonstrated that radiotherapy reduced the risk of local failure by 4.7 times in a clinical study involving 58 patients [70].

Radiotherapy has also been used as palliative care treatment in ACC cases associated with bone metastasis [4, 29, 71]. Despite a certain difficulty in monitoring some parameters, it seems that ionizing radiation treatment was able to reduce metastatic size and symptoms in 57% of cases [27].

Radiation treatment as an adjuvant therapeutic option has been described to significantly reduce recurrence rates, thus suggesting a significant therapeutic potential. In particular, Fassnacht et al. reported that the probability of recurrence risk reduction was significantly higher in a group of patients treated with 45–55 Gy for five weeks after surgery, than in patients who did not undergo radiotherapy (79% versus 12%) [72].

Existing data regarding radiotherapy efficacy in ACC indicate that this treatment should be taken into consideration only after having carefully evaluated the clinical picture of every patient [27, 29]. In particular, radiotherapy is recommended when microscopic tumor residues are detectable after surgery (R1), whereas those patients who exhibit macroscopically visible residual tumours (R2) are advised to undergo a second operation. Radiotherapy is suitable also in cases where residual tumour dimensions are not known (RX) and when recurrence risk is high. Finally, patients with advanced disease and those with a stage III tumour with local lymph node invasion and no distant metastasis may benefit from adjuvant radiotherapy [27, 73].

Currently, no general guidelines exist for radiotherapy use in patients who have undergone complete tumour removal (R0), although this treatment is not usually not recommended when tumour dimensions are ≤8 cm. Instead, it can be considered for tumours with greater dimensions, blood vessels invasion (V1), and a Ki-67 index ≥ 20%, which are associated with a high recurrence risk [27, 65, 74].

Based on clinical observations, treatment planning should be individualized on the basis of patient characteristics however radiotherapy as adjuvant therapy should start as soon as possible, within 3 months from surgery [27, 65]. The optimum radiation protocol has not yet been defined in fact the highest total dose reported was 60 Gy, administered in daily fractions of 1.5–1.8 Gy over 5–7 weeks [75] however many studies recommended lower doses, ranging from 20 Gy to 55 Gy [27]. In general, radiotherapy treatment on tumors should be carried out for 5 to 6 weeks at doses of 1.8–2.0 Gy *per* fraction, with total amounts ranging from 40 Gy to 50/60 Gy [27, 75].

Combined treatment based on the association between radiotherapy and cytotoxic drugs, such as mitotane, is currently under investigation. Some *in vitro* studies, in fact, reported an inhibitory effect of mitotane *plus* ionizing radiations on ACC cell lines [76, 77]. A recent study by Salboch et al. [70] evaluated the effect of mitotane administration in ACC patients and found no differences in response rates in the surgery group or surgery and radiotherapy group after mitotane treatment (25% versus 20%, resp.) while other authors argue that radiotherapy efficacy might be ameliorated by concomitant administration of mitotane or other chemotherapeutic agents [78, 79]. According to some authors, mitotane treatment in association with radiotherapy is recommended for patients who underwent R1 and RX resection. However, mitotane doses should be <3 g/d, in order to prevent sever hepatic toxicity; moreover, levels of GOT, GPT, and bilirubin should be monitored every 2/3 weeks [74].

## 6. Targeted Therapies

Recent advances in the understanding of genetic alterations involved in ACC onset and progression led to the identification of several potential molecular targets for selective therapy of ACC. Until recently, the main genetic modifications discovered in ACC cases involved oncosuppressor genes, such as *TP53*, *CDKN1C*, *MEN1,* and *CDKN2A*, and oncogenes such as *IGF2*, *RAS* and *CTNNB1*. Currently many other genes are under investigation in order to understand their usefulness for the development of new therapeutic strategies [67, 69, 80, 81].

**Insulin-Like Growth Factor-2 Pathway**

Overexpression of insulin-like growth factor-2 (IGF-2) represents the most important molecular event identified in ACCs, which occurs in more than 90% of cases [6, 82]. IGF-2 hypersecretion translates in an unchecked cell proliferation, due to the activation of PI3K/Akt/mTOR pathway through IGF-1R [83, 84]. Preclinical studies on cell and xenograft models demonstrated that NVP-AEW541, a small molecule inhibitor, and IMC-A12, a fully human monoclonal antibody, both targeting IGF-1R, were able to inhibit IGF-2 downstream pathway and to reduce cell proliferation. Moreover, the association of these molecules with mitotane strongly inhibited tumor growth in a synergistic way [71, 83].

Recently, two phase I studies demonstrated the efficacy of figitumumab and OSI-906 in inducing a partial tumor response in 57% and 33% of patients, respectively [71, 85, 86]. Figitumumab is an anti-IGF-1R monoclonal antibody, and OSI-906 is a small molecule tyrosine kinase inhibitor directed against IGF-1R. Moreover, an international phase III trial is currently in progress, in order to evaluate the feasibility and efficacy of OSI-906 for treatment of patients with ACC should end in 2013 [87].

Recent studies demonstrated the association between *IGF2* overexpression, mTOR hyper-activation and reduced expression of miR-99a and miR-100, whose function thus appeared to be the inhibition of these factors [88]. The role of mTOR in malignant tumors onset has been established by several studies, thus highlighting its important as a potential therapeutic target for ACC [71, 89]. The most important inhibitors of mTOR are rapamycin (sirolimus) and its derivatives everolimus (RAD001) and temsirolimus (CCI-779) [89]. A recent study demonstrated that pharmacologic inhibition of mTOR signaling by everolimus greatly reduced adrenocortical tumor cell growth both *in vitro* and *in vivo*, also confirming the importance of microRNA regulation of IGF-2/mTOR signalling cascade [88]. Moreover, a phase I clinical trial evaluating the effect of temsirolimus in combination with the anti-IGF-1R recombinant monoclonal antibody cixutumumab in advanced malignancies demonstrated a tumor reduction in 4 of 10 patients with ACC [71, 90].

**Angiogenesis**

Considering the great importance of angiogenesis and neovascularization for tumor proliferation and migration, new therapeutic strategies have been conceived. These approaches either prevent new blood vessel formation or disrupt existing tumor vasculature. In ACC, vascular endothelial growth factor (VEGF) is over-expressed and its levels appear to decrease after tumor removal, thus confirming its role in ACC growth and its importance as an efficient therapeutic target [57, 69, 71].

Despite the expanding interest in this field, clinical trials employing antiangiogenic drugs are quite inefficient; moreover, the results obtained to date are discouraging. A recent study involving 10 patients with advanced ACC who were treated with the monoclonal VEGF antibody bevacizumab (Avastin) in combination with the oral pro-drug capecitabine as salvage therapy reported no objective response or stable disease. Moreover, this therapeutic regimen caused severe side effects which required treatment suspension in two cases [91]. Instead, a single case report described instead a partial response in a 40-year-old patient with advanced chemoresistant ACC taking 200 mg/d thalidomide [92].

More encouraging results have been obtained in clinical studies employing small-molecule tyrosine kinase inhibitors targeting VEGFR, such as sorafenib and sunitinib [71]. Sunitinib has been found to induce a strong adrenal toxicity in animal models, and a partial response to this treatment has been reported in a single patient with metastatic ACC, after failure of mitotane-based chemotherapy creating the necessary conditions for the beginning of a phase II trial with sunitinib as monotherapy for refractory ACCs [71, 93, 94]. The first demonstration of sorafenib efficacy in ACC treatment derives from a phase I trial employing sorafenib plus the farnesyltransferase inhibitor tipifarnib. This trial reported stable disease in two patients with advanced ACC [95]. Moreover, a single-case report described a sustained regression of metastatic lesions associated with a stage IV ACC after sorafenib administration [96]. Recently, a phase II study investigating the effects of sorafenib in combination with metronomic paclitaxel was conducted, in order to evaluate both the efficacy of sorafenib treatment and the potential of metronomic therapy in inhibiting tumor growth. Despite *in vitro* data suggesting that sorafenib was able to reduce viability of H295R cells, treatment of patients with advanced ACC was ineffective; moreover, paclitaxel administration did not increase the effect of sorafenib *in vitro* [71, 93, 97].

The antiangiogenic effect of rapamycin has been demonstrated *in vitro*, although the clinical data currently available is insufficient [57, 71]. Nevertheless, in a recent study, Gangadhar et al. observed a partial response in a patient with ACC treated with sirolimus and sunitinib in combination [98].

Finally the significance of heparanase-1 in ACC angiogenesis has recently been highlighted, suggesting that this protein could represent selective target treatment of ACC [99].

**Tyrosine Kinase Inhibitors (TKIs)**

The identification of molecular targets for ACC is often achieved through microarray and transcriptome analyses, which allows the identification of some signalling pathways that are disrupted in this neoplasia [57, 84, 100]. Many of these pathways are hyperactivated by the overexpression of growth factors, such as IGF-II, EGF, and FGF, which is a frequent event occurring in ACC. Thus novel therapeutic strategies are thus based on the inhibition of protein kinases involved in signal transduction, especially receptor tyrosine kinases. In addition to IGF-1R and VEGFR inhibitors, mentioned above, there has been a substantial investment in the development of inhibitors of other tyrosine kinase receptors, such as EGFR and PDGF (platelet-derived growth factor) receptor [57, 71].

*In vitro* studies demonstrated that suramin, an anti-parasitic drug known to inhibit the binding of growth factors (e.g., EGF, PDGF, TGF-*β*, FGF-*β*) to their receptors, was able to antagonize the ability of these factors to stimulate tumor cells' proliferation and reduced cortisol secretion [101]. Further studies, however, showed that the effect of suramin was partial and that the side effects were extremely severe. Therefore this drug is not currently used for ACC treatment [102].

Recently, a clinical study conducted on ten patients with advanced ACC treated with erlotinib, an EGFR inhibitor, in combination with gemcitabine reported very limited to no efficacy of this therapeutic regimen [103]. Similarly, Samnotra et al. observed a 0% response rate in a cohort of 19 patients with pathologically confirmed unresectable ACC treated with gefitinib (Iressa ) as a second-line monotherapy [104]. The reasons of this therapeutic failure might be that EGFR is over-expressed in approximately 76% of ACC; however no mutations were found in the *EGFR *gene. In addition, EGFR expression does not represent a useful prognostic factor, questioning its real therapeutic value [57, 93, 105]. Similar observations were made regarding PDGFR, whose expression does not seem to be altered in ACC cases [57]. Consistently with that, a phase II study involving 4 patients with advanced ACC treated with oral imatinib mesylate, a PDGFR inhibitor, reported disease progression in three cases. In one case the side effects were so severe that treatment was suspended [106].

**MDR/P-Glycoprotein**

It has long been known that ACC is a chemoresistant tumor, and this fact seems to be related to the overexpression of the multidrug resistance protein MDR-1 (P-glycoprotein, Pgp), which is an ATP-dependent drug efflux pump [57, 58, 93]. Moreover, some MDR-1-independent mechanisms seem to be involved in ACC drug resistance, and excision repair cross-complementing group 1 (ERCC1) is also shown to play a role in the resistance to platinum-based treatment [57, 107].

So far, several compounds which can interfere with MDR-1 function have been so far identified. The MDR-1 inhibitor verapamil has been shown to improve chemo-sensitivity in leukemic and ovarian cancer, although its effectiveness in ACC has not been proven yet [70, 108]. Second- and- third generation MDR-1 modulators, including D-verapamil, valspodar (PSC833), an analogue of cyclosporine D, and tariquidar (XR9576), a P-glycoprotein drug efflux pump inhibitor, have been developed in order to strengthen the cotreatment with cytotoxic agents. However, the effect of these compounds is rather unsatisfying [57, 71]. Moreover, results from a preclinical study employing primary human ACC cells treated with doxorubicin and vincristine in association with Pgp antagonists verapamil, cyclosporine A, and its analogue SDZ PSC833 indicated that the resistance to chemotherapy in ACC is mediated by mechanisms other than Pgp [109].

Despite the results of clinical trials obtained on a small numbers of patients with metastatic disease seem discordant from the effects obtained *in vitro*, further studies are necessary to prove the efficacy of inhibitors of Pgp.

The chemosensitizing effect of mitotane has also been investigated. *In vitro* studies demonstrated that clinically achievable concentrations of *o,p'*-DDD could increase drug accumulation, due to the inhibition of Pgp-mediated drug efflux [58]. However, clinical trials employing doxorubicin, vincristine, and etoposide in combination with mitotane failed to demonstrate the effectiveness of this treatment [110, 111].

**PPAR-γ Antagonists**

The nuclear receptor PPAR-*γ* is highly expressed in the normal and neoplastic adrenal cortex and is implicated in the regulation of the IGF-2/IGF-1R signalling pathway, by inhibiting Akt activation [112, 113]. The use of PPAR-*γ*antagonists is complicated by the observation that thiazolidinediones (TZDs) can induce severe adverse effects, particularly on the cardiovascular system [113]; nevertheless these compounds were shown to be able to inhibit cell proliferation in ACC cell lines and xenograft models [112–116].

The molecular mechanism underlying the anti-proliferative and prodifferentiating effects of rosiglitazone, a member of the TZD class, in ACC has not been completely elucidated. However it has been demonstrated that both PPAR-*γ*-dependent and -independent pathways were activated by this drug, leading to growth arrest, cell death and decreased *VEGF* expression, which could be involved in the reduction of tumor infiltration and neovascularization [112, 114, 116].

**Wnt/*β*-catenin pathway**

The involvement of Wnt/*β*-catenin pathway in adrenocortical tumorigenesis is supported by the observation that in many adrenocortical tumors, both benign and malign, an accumulation of *β*-catenin protein was noticed [8, 69, 117]. Constitutive activation of this protein seems to be a main event leading to adrenocortical carcinogenesis; moreover nuclear localization of *β*-catenin represents a predictive factor for a worse prognosis in ACC cases [8, 117, 118].

Preclinical *in vitro* studies evaluated the effect of PKF115-584, a small molecule inhibitor of the T-cell factor (Tcf)/*β*-catenin complex, on *β*-catenin-dependent transcription and proliferation in H295R ACC cells, harbouring mutations in *CTNNB1* gene. Treatment with PKF115-584 inhibited cell proliferation and induced apoptosis in a dose-dependent way in H295R cells, but not in HeLa cells, thus indicating that targeting the Wnt/*β*-catenin pathway might be useful in the treatment of adrenocortical tumors [119]. CWP232291 is a compound which is able to promote *β*-catenin degradation. It also exhibited potent growth inhibitory activity in several multiple myeloma cell lines and a phase I clinical study of CWP232291 in patients with relapsed or refractory acute myeloid leukemia is currently ongoing (http://www.clinicaltrials.gov/, trial ID NCT01398462) [120]. However, no clinical data is available at present concerning ACC; moreover, despite the great potential of Wnt/*β*-catenin inhibition for ACC treatment, the involvement of these factors in many fundamental cellular processes should always be considered during treatment planning, in order to prevent severe toxicity and adverse effects [120, 121].

**Steroidogenic Factor-1**

Steroidogenic factor-1 (SF-1) is a nuclear receptor involved in adrenal and gonadal development, steroidogenesis, and reproductive axis regulation. *SF-1* gene is frequently amplified and over-expressed in pediatric ACCs, whereas in adult carcinomas chromosomal abnormalities in chromosome 9 have been noticed. Moreover, patients showing higher levels of SF-1 expression seem to have a worse prognosis compared with those who express lower levels of this factor [122, 123]. It has previously been found that increased SF-1 dosage stimulates proliferation, decreases apoptosis of human adrenocortical cells, and induces ACTs in transgenic mice [124]. *In vitro* studies performed on H295R cells demonstrated that SF-1 gene silencing strongly affected TGF-*β* and Wnt/*β*-catenin signalling, suggesting the existence of a crosstalk between these pathways. Moreover, SF-1 knockdown induced a significant reduction of proliferation rate in treated cells compared to control cells and a reduction of cells in the S-phase [124, 125].

Recently, synthetic SF-1 inverse agonists have been identified and tested in ACC cell lines. Two members of the alkyloxyphenol class, AC-45594 and OOP, were shown to inhibit proliferation of H295R and SW-13 cells through a mechanism which did not seem to be selective for SF-1. On the contrary, members of the IsoQ class, SID7969543 (IsoQ A), and the number 31 and 32 compounds, selectively inhibited cell proliferation in conditions of increased SF-1 expression, strongly suggesting that the IsoQ drugs selectively target the activity of SF-1-related genes, thus representing a potential new class of compounds to be used in ACC treatment [126].

**Gene Therapy and Immunotherapy**

The use of gene therapy is an evolving approach for cancer treatment; nevertheless, poor results have so far been obtained in ACC. The aim of gene therapy would be to re-activate oncosuppressor genes and/or to inhibit oncogenes, whose expression is deregulated during tumor progression. The systemic administration of antisense oligonucleotides would represent an interesting approach for ACC therapy; moreover, an *in vitro* study demonstrated that a suicide vector, in which the herpes simplex virus thymidine kinase (HSV-TK) gene was driven by the CYP11B1 promoter with a P450scc enhancer element, was able to increase chemosensitivity in Y1 mouse adrenocortical cancer cells [57, 127].

Immunotherapy is a therapeutic approach based on the stimulation of the immune response against cancer cells. Recently, immunotherapy using dendritic cells has been proven to be safe and effective in inducing antitumor immune responses leading to tumor regression [128]. In a study conducted on two patients with metastasized hypersecretory ACC, the vaccination with autologous dendritic cells was able to induce antigen-specific Th1 immunity. However, no clinical advantage was observed [93, 129]. The main limitation of this therapy is the difficulty in identifying specific tumoral antigens; however some interesting targets for a specific immunotherapeutic approach in ACC might be represented by steroidogenic factor 1, surviving and steroidogenic acute regulatory protein (StAR) [94, 130].

**Estrogen Pathways**

Estrogens are produced by the enzyme aromatase using androgens as substrate, and Barzon et al. have shown that ACC are characterized by aromatase overexpression [131]. Then, it is possible that in ACC patients despite normal circulating estrogen levels a higher local estrogen production can occur. The classical mechanisms of estrogen action are mainly mediated by two members of the nuclear receptor superfamily, the estrogen receptor (ER) *α* and *β* [132]. In ACCs also has been demonstrated a differential expression of ERs [131, 133]. Another study demonstrated that hydroxytamoxifen controls proliferation of H295R cells by decreasing ER*α* and upregulating ER*β* expression levels causing an increase in the expression of the pro-apoptotic factor FasL [134]. Very recent studies using H295R cells, demonstrated a central role for ER*α* in both E2- and IGF-II-dependent cell proliferation, suggesting that targeting this receptor could be effective in controlling ACC growth. The authors have demonstrated that IGFII is capable of activating ER*α* through phosphorylation in an estrogen-independent manner, suggesting that in ACC ER*α* can be activated also by IGF-II. In addition, exploiting the ability of H295R cells to generate xenografts in athymic nude mice, this preliminary data demonstrated an hypothetical role of the selective estrogen receptor modulator (SERM) tamoxifen to control ACC growth *in vivo* [135].

## 7. Summary and Conclusion

Despite advances in the biology of ACC, prognosis remains very poor. Although exciting results have been obtained in lab regarding new therapies, their application in clinical practice has been somewhat disappointing. The organization in the international study groups is certainly an important goal for research in the context of a rare malignancy. So far this has led to the conclusion of the FIRM-ACT and ADIUVO equipment. An early diagnosis is fundamental for the prognosis and for an improvement of the survival rate of patients affected by ACC. Recent contributions by transcriptomics and proteomics have identified specific biomarkers able to discriminate between more aggressive and less aggressive forms. However, the aggressiveness of this tumor still requires efforts in the identification of prognostic and therapeutics markers to use not only in clinical settings but also for the design of new specific drugs. Considering the biological heterogeneity of this malignancy, new therapeutic strategies, such as a target therapy, could be the future in ACC treatment.

## Figures and Tables

**Table 1:** Staging systems of adrenal cortical carcinoma (ACC) according to the criteria of the Union Internationale Contre Cancer (UICC) 2004 and the European Network for the Study of Adrenal Tumors (ENSAT) 2008.

Stage	UICC/WHO 2004	ENSAT 2008
I	T1, N0, M0	T1, N0, M0
II	T2, N0, M0	T2, N0, M0
III	T1-2, N1, M0 T3, N0, M0	T1-2, N1, M0 T3-4, N0-1, M0
IV	T1–4, N0-1, M1 T3, N1, M0 T4, N0-1, M0	T1–4, N0-1, M1

T1: tumor ≤5 cm; T2: tumor >5 cm; T3: tumor infiltration into surrounding tissue; T4: tumor invasion into adjacent organs or venous tumor thrombosis; N0: no positive lymph nodes; N1: positive lymph node(s); M0: no distant metastasis; M1: distant metastasis.

**Table 2:** Treatment protocols employed in the FIRM-ACT study.

Berruti and *coll*. protocol (EDP/M)	Every 28 days		
	(i) Day 1	40 mg/m^2^	Doxorubicin
	(ii) Day 2	100 mg/m^2^	Etoposide
	(iii) Day 3, 4	100 mg/m^2^	Etoposide + 40 mg/m^2^ cisplatin
	(iv) Daily		Mitotane with a blood level 14–20 mg/L

Khan and *e coll.* protocol (Sz/M)	Every 21 days		
	(i) Day 1–5	1 g	Streptozotocin
	(ii) Subsequently	2 g	Streptozotocin
	(iii) Daily		Mitotane with a blood level 14–20 mg/L
